# Expression of an estrogen-regulated variant transcript of the peroxisomal branched chain fatty acid oxidase ACOX2 in breast carcinomas

**DOI:** 10.1186/s12885-015-1510-8

**Published:** 2015-07-17

**Authors:** Sunniva Stordal Bjørklund, Vessela N. Kristensen, Michael Seiler, Surendra Kumar, Grethe I. Grenaker Alnæs, Yao Ming, John Kerrigan, Bjørn Naume, Ravi Sachidanandam, Gyan Bhanot, Anne-Lise Børresen-Dale, Shridar Ganesan

**Affiliations:** 1 Department of Genetics, Institute for Cancer Research, Oslo University Hospital Radiumospitalet, Montebello, 0310 Oslo, Norway; 2 Rutgers Cancer Institute of New Jersey, 195 Little Albany Street, New Brunswick, NJ 08903 USA; 3 Department of Molecular Biology & Biochemistry, Rutgers University, Piscataway, NJ 08854 USA; 4 Department of Oncological Sciences, Mount Sinai School of Medicine, New York, NY 10029 USA; 5 The K.G. Jebsen Center for Breast Cancer Research, Institute for Clinical Medicine, Faculty of Medicine, University of Oslo, Oslo, Norway; 6 Department of Clinical Molecular Biology and Laboratory Science (EpiGen), Akershus University hospital, Division of Medicine, 1476, Lørenskog, Norway; 7 Center for Advanced Genomic Technology, Boston University, Boston, MA 02215 USA; 8 Department of Oncology, Oslo University Hospital, Radiumhospitalet, Oslo, Norway

**Keywords:** Breast cancer, Fatty acid oxidation, Gene transcription, Steroid hormone receptor, Tumor marker

## Abstract

**Background:**

Alternate transcripts from a single gene locus greatly enhance the combinatorial flexibility of the human transcriptome. Different patterns of exon usage have been observed when comparing normal tissue to cancers, suggesting that variant transcripts may play a role in the tumor phenotype.

**Methods:**

Ribonucleic acid-sequencing (RNA-seq) data from breast cancer samples was used to identify an intronic start variant transcript of Acyl-CoA oxidase 2, ACOX2 (ACOX2-i9). Difference in expression between Estrogen Receptor (ER) positive and ER negative patients was assessed by the Wilcoxon rank sum test, and the findings validated in The Cancer Genome Atlas (TCGA) breast cancer dataset (BRCA). ACOX2-i9 expression was also assessed in cell lines using both quantitative reverse transcriptase-polymerase chain reaction (qRT-PCR) and Western blot analysis. Knock down by short hairpin RNA (shRNA) and colony formation assays were used to determine whether ACOX2-i9 expression would influence cellular fitness. The effect of ACOX2-i9 expression on patient survival was assessed by the Kaplan-Meier survival function, and association to clinical parameters was analyzed using a Fisher exact test.

**Results:**

The expression and translation of ACOX2-i9 into a 25 kDa protein was demonstrated in HepG2 cells as well as in several breast cancer cell lines. shRNA knock down of the ACOX2-i9 variant resulted in decreased cell viability of T47D and MDA-MB 436 cells. Moreover, expression of ACOX2-i9 was shown to be estrogen regulated, being induced by propyl pyrazoletriol and inhibited by tamoxifen and fulvestrant in ER+ T47D and Mcf-7 cells, but not in the ER- MDA-MB 436 cell line. This variant transcript showed expression predominantly in ER-positive breast tumors as assessed in our initial set of 53 breast cancers and further validated in 87 tumor/normal pairs from the TCGA breast cancer dataset, and expression was associated with better outcome in ER positive patients.

**Conclusions:**

ACOX2-i9 is specifically enriched in ER+ breast cancers where expression of the variant is associated with improved outcome. These data identify variant ACOX2 as a potential novel therapeutic biomarker in ER+ breast tumors.

**Electronic supplementary material:**

The online version of this article (doi:10.1186/s12885-015-1510-8) contains supplementary material, which is available to authorized users.

## Background

Breast cancer is the most common form of cancer among women worldwide. It is a greatly heterogeneous disease with respect to prognosis, treatment response, and patient outcome. In the past decade, clinically diverse subclasses based on the expression profiles of specific sets of genes, have been identified [1–3]. Different methods have identified at least four stable subgroups of cancer which correspond with the following clinical characteristics 1. ER- positive, HER2-nonamplified and low-grade (Lum-A), 2. ER-positive, HER2-nonamplified and high-grade (Lum-B), 3. ER-, PR-, and Her2-negative (Basal-like), 4. ERBB2/Her2-amplified (HER2 enriched). Recently Curtis et al. proposed a further subdivision into ten distinct molecular groups based on both expression and copy number [4]. This emphasizes the complexity of breast cancer, and the need for robust classification and biomarkers for the existing subtypes in order to make optimal treatment decision for each individual patient.

Alternate transcripts from alternative splicing of a single gene locus increase the number of gene products encoded by the human genome. It is estimated that 90 % of all multi-exon genes are subjected to some form of alternative splicing [5]. Alternate promoter usage and post-transcriptional processing of mRNAs can give rise to functionally distinct protein isoforms. There is increasing evidence linking aberrant and alternative transcription to cancer. However, to date, very little is known about the mechanisms involved, or whether alternate transcripts are a driving force or the result of cancer progression [6].

Cancer cells are known to undergo several changes in metabolism, which render them more efficient at producing macromolecules necessary for growth and proliferation. Numerous studies have focused on the metabolic switch to aerobic glycolysis, a process that may not be as efficient for ATP production, but which is highly responsive to changes in the cell’s need for energy and macromolecules used for building mass [7]. This form of glycolysis also supports lipid synthesis and directs amino acids to protein synthesis, both processes necessary for growth and proliferation [8]. Recent studies have focused on fatty acid oxidation (FAO), showing that increased FAO facilitates survival of mammary epithelial cells as they detach from the extra cellular matrix [9]. Additionally, the expression of genes involved in increased FAO have been shown to be associated with poor prognosis in breast cancer [10].

**Fig. 1:**
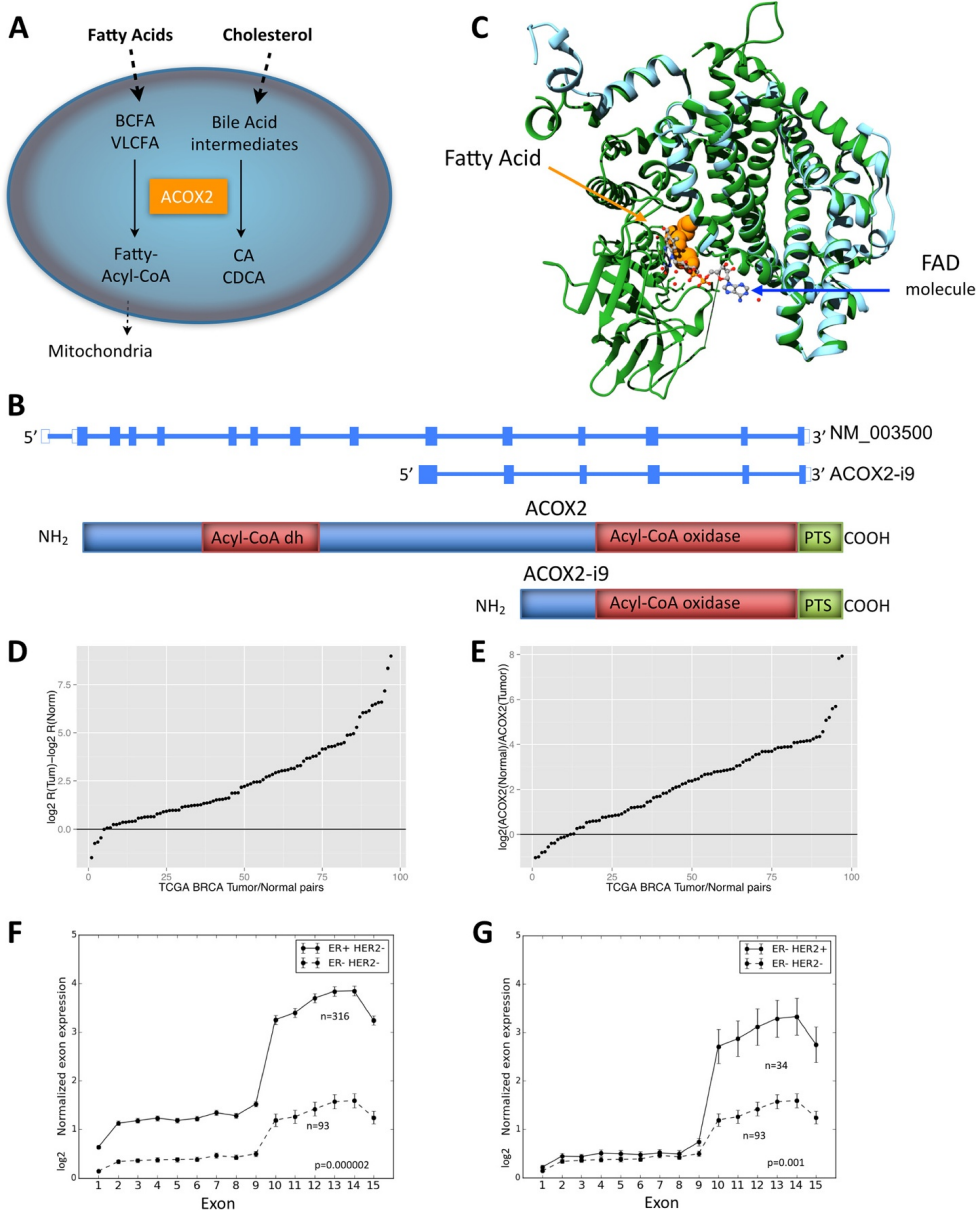
*ACOX2 expression in TCGA BRCA cohort*. ACOX2 is involved in the oxidation of very long chain fatty acids, VLCFA, and branched chain fatty acids, BCFA, and in the synthesis of bile-acid precursor molecules as schematically illustrated in Fig. 1a. The ACOX2 intronic variant, ACOX2-i9, is initiated just upstream of exon 10 of the full-length transcript (**b**). The translated protein retains the Acyl-CoA oxidase domain, and the Preoxisomal Targeting Signal, but lacks the fatty acid binding domain of the full-length protein. 1**c** shows a model of ACOX2-i9 aligned with 2DDH (Rat ACOX2). The template 2DDH is colored green and the model (i9) is colored cyan. The fatty acid is depicted as orange colored spheres and the FAD molecule (and water depicted as small red spheres) is depicted as ball-stick and colored by element. The difference in Log2 R(ACOX2-i9/ACOX2) Tumor – log2 R Normal in 87 tumor/normal pairs from the TCGA BRCA dataset are shown in **d**, see Methods for details. Values > 0 indicate that the Ratio of ACOX2-i9/ACOX2 is higher in the tumor. **e** shows log2 expression of ACOX2 in Normal/Tumor. ACOX2 is expressed at higher levels in the Normal sample when the log2 ratio >0. Normalized log2 RPKM expression of each exon of ACOX2 in Her2 negative background separated by ER status are shown in **f**, and in ER negative patients separated by Her2 status (**g**)

ACOX2 is the rate limiting enzyme in the β-oxidation of branched, long chain fatty acids and in the synthesis of Bile-acid precursor molecules [11] (Fig. 1a). A variant transcript of ACOX2 has been detected in hepatocellular carcinoma (HCC) where it was suggested to play a role in tumor progression [12].

Using RNA-seq data, we found that this ACOX2 variant, ACOX2-i9, is present in a subset of human breast cancers. We further demonstrated that this variant is translated into a detectable protein in breast cancer cell lines, and that knockdown of the variant led to decreased cell growth and viability. The TCGA breast cancer dataset and an independent cohort of 113 tumors from patients with long term follow up was used to investigate expression of the variant in clinical samples as well as to study it’s association with clinical outcome.

## Methods

### Cell lines

HeLa, Hek-293 T, HepG2, Mcf-7, MDA-MB 231, MDA-MB 436, MDA-MB 468, and T47D cells were obtained from ATCC and kept in recommended growth media at 37 degrees, supplemented with 5 % CO_2_.

### qRT-PCR

RNA was extracted from cells using the Trizol reagent according to the manufacturer’s instructions. Complementary DNA was transcribed using Superscript II from Invitrogen for cell lines and High-Capacity cDNA Reverse Transcription Kit (Applied Biosystems) for patient samples. Real-Time PCR was carried out on the Mx3005p QPCR system using SYBR green dye for detection. Transcript levels were assayed in triplicates and normalized to *GAPDH* mRNA expression. Primer efficiency was assayed for all pairs using a standard dilution curve, and relative expression levels were calculated using the method suggested in [13]. Primers were designed using Primer3 software and were as follows: ACOX2 forward 5’GCAAAGGTCCTGGACTACCA3’, reverse 5’CCAGGGGACATCTGAGTCT3’. ACOX2-i9 forward 5’ACAGGGTTGGTCCCTATGGT3’, reverse 5’AGGTCAGGTGCGGTGAGATA3’. The same primers were used for qRT-PCR, conventional RT-PCR, and Sanger sequencing of patient samples.

### Cloning and constructs

RNA from HepG2 cells was isolated using the Trizol reagent, and cDNA was synthesized using Superscript II from Invitrogen. PCR was carried out with the Pfu ultra enzyme (Sigma). Full length ACOX2, and ACOX2-i9 were cloned into the TOPO-pcDNA3.1-V5/His vector (Sigma) using the following primers; ACOX2 forward 5’CACCATGGGCAGCCCAGTGCA 3’, ACOX2-i9 forward 5’ CACCATGAGTAGATGCTCAGTA 3’, reverse (same for both) 5’ TAGCTTGGATCTCCAACTTTG 3’ and both constructs were confirmed by sequencing.

### shRNAs and stable knock down cells

shRNA constructs in the pLKO.1 Lentiviral vector were purchased from Sigma. Viral packaging vectors psPAX2 and pMD2.G were obtained from Addgene (plasmids 12260 and 12259). Recommended protocol from Addgene was followed. Briefly; Hek-293 T cells were transfected with three plasmids, psPAX2, pMDG.2, and either empty pLKO.1 vector (control), pLKO.1 vector with shRNA targeting the N-terminal region of ACOX2 (TRCN0000046214 (N) and TRCN0000046215 (N’), or shRNA targeting the C-terminal region (TRC0000046217 (C) and TRCN0000046216 (C’) using the Fugene 6 transfection reagent. Viral particles were harvested after 48 and 72 h, and were used to infect T47D and MDA-MB 436 cells in media containing 8ug/ml polybrene. Cells were selected using RPMI1640/DMEM:F12(1:1) media supplemented with 2,5 ug/ml Puromycin for 5 days and kept under selective pressure. Knockdown was confirmed by Western blotting.

### Western Blot

Protein lysate was extracted using NETN buffer (20 mM Tris (pH 8.0), 150 mM NaCl, 1 mM EDTA, 0.5 % NP40, 1x Protease inhibitor cocktail (Roche)). 30–40 ug protein, optimized for each cell line, was loaded onto an Any-kD SDS Polyacrylamide gel from Biorad, transferred to a Nitrocellulose-membrane and probed with the C-terminal monoclonal ACOX2 antibody from Sigma (SAB1404576) or with a Tubulin antibody (Invitrogen).

### In-vitro transcription and translation

In-vitro expression of ACOX2-i9 was carried out using the human In vitro protein expression kit for DNA templates (Pierce) using 1 ug pcDNA3.1-V5/His-ACOX2-i9 vector and following the manufacturers instructions. Expression was assayed by western blotting using the C-terminal ACOX2 antibody.

### Treatment of cell lines with selective estrogen receptor modulators (SERMs)

T47D, Mcf-7, MDA-MB 436, and HepG2 cells were maintained under normal growth conditions and supplemented with vehicle (EtOH/DMSO), 100nM 4-Hydroxytamoxifen (4-OHT, tamoxifen) (HepG2- 200nM), 100nM fulvestrant (ICI 182,780), or 1-100nM propyl pyrazoletriol (PPT) as indicated for 48 h. For estrogen depletion, cells were kept in Phenol-Red-free RPMI1640/DMEM/DMEM:F12(1:1) supplemented with 10 % Charcoal stripped FBS for 72 h.

### Colony formation assay

T47D stable cell lines were plated 200 cells per well in 6 well plates. MDA-MB 436 cells were plated 500 cells per well in 6 well plates. All experiments were carried out in triplicates and replicated at least 3 times. Cells were kept in normal growth conditions, supplemented with 2.5 μg Puromycin for 15 days. Cells were fixed by Methanol fixation, and stained with 0.5 % Crystal Violet. Colonies containing 50 cells or more were counted as colonies.

### Datasets

A. 37 tissue samples from the Cancer Institute of New Jersey (CINJ) in NJ, USA and 16 tissue samples from Oslo University Hospital, Radiumhospitalet, Norway, Norway underwent RNA extraction using the Trizol reagent per the manufacturer’s protocol. We followed the standard Truseq protocol recommended by Illumina for library preparation, and sequencing was carried out using the Illumina Genome Analyzer IIx or the Illumina HiSeq 2000 at the Mount Sinai School of Medicine (MSSM). Raw sequence data is available from the Sequence Read Archive using accession number SRA057220. 29 bp single end reads were aligned using TopHat version 2.0.9 against the human reference genome (GRCh37.72). Cufflinks-2.2.0 was used to assemble and estimate transcript abundance using the annotation file provided in the package. Cuffdiff was used to assess differential expression between ER positive and ER negative samples. The CummeRbund R package was used to further explore, visualize and analyze result files obtained from cuffdiff.
B. A total of 113 breast tumor tissue samples from the MicMa cohort [14] underwent RNA extraction using the Trizol reagent per the manufacturer’s protocol, and PCR was performed to determine whether the variant could be detected. Long-term follow up data from this dataset was used for survival analysis.
C. Clinical and RNAseq data from the publically available TCGA dataset from 846 breast cancer patients, including all the 97 samples with a matched normal sample was obtained and used for analysis of ACOX2-i9 expression in tumor versus normal samples, and of expression in groups of different clinical compositions. ER and Her2 status were determined by immunohistochemistry and annotated in the clinical file. A total of 87 tumor/normal pairs with clearly known ER status were used for this analysis.

Expression of the two different ACOX2 transcripts were calculated based on the following; The ACOX2 gene has 15 exons. Let L0 be the sequence length of exons 1–15 and let L1 be the sequence length of exons 10 through 15. Let r0 be the sum of reads mapped to exons 1–9 and r1 be the sum of reads mapped to exons 10–15. Note that these are raw reads and not log transformed or processed into RPKM.

We will assume that the reads per unit nucleotide are uniform for both the full transcript and the i9 transcript (i.e., that these are the only two variants in the sample). In this case, the number of reads assignable to the full transcript are:

$$ \mathrm{F}0 = \left(\mathrm{r}0 \times \mathrm{L}0\right)/\left(\mathrm{L}0-\mathrm{L}1\right), $$

and the number of reads assignable to the i9 transcript are:

$$ \mathrm{F}1=\mathrm{r}1\hbox{-} \mathrm{F}0\times \mathrm{L}1/\mathrm{L}0. $$

We then define the fraction R, which estimates the ratio of the mRNA level of the i9 transcript to the full transcript as:

$$ \mathrm{R} = \left(\mathrm{mRNA}\ \mathrm{Expression}\ \mathrm{of}\ \mathrm{i}9\ \mathrm{transcript}\right)/\left(\mathrm{mRNA}\ \mathrm{Expression}\ \mathrm{of}\ \mathrm{full}\ \mathrm{transcript}\right) $$

From this it follows that:

$$ \mathrm{R} = \left(\mathrm{F}1/\mathrm{L}1\right)/\left(\mathrm{F}0/\mathrm{L}0\right) $$

log2 RPKM pr exon was used to plot all exons in the ACOX2 locus from all patients in the TCGA cohort. The difference in R in distinct clinical subgroups was assessed by a Wilcoxon rank sum test.

### Survival analysis

Kaplan-Meier survival curves were calculated using the Survival package in R. 6 of the 113 patients in the cohort did not have survival data and were excluded from this part of the analysis. For statistical analysis the tumors were categorized as ACOX2-i9 positive if a band was detected after 35 cycles of PCR, or ACOX2-i9 negative if no band was detected. For test of association to clinical parameters, a Fisher exact test was used. Analysis was conducted in the R environment using R version 3.1.2.

### Ethics statement

Use of the samples from Oslo University Hospital was approved by the Norwegian Regional Committee (REC) for Medical and Health Research Ethics (REC South East, reference numbers S97103 and 429–04148), all patients were informed and have declared written informed consent that their samples are used for research. Samples from Rutgers Cancer Institute of New Jersey were de-identified patient samples collected under a tissue banking protocol and approved for use in this study by The Rutgers Health Sciences New Brunswick/Piscataway Institutional Review Board, number 0220080121. Individual patient consent for the use of these patient samples was not required.

## Results

### A variant of ACOX2 was identified in a subset of breast carcinomas, and its presence was validated in the TCGA BRCA dataset

In a genome wide screen aimed at identifying alternative transcripts in breast tumors using RNA-seq, the presence of an alternative mRNA transcribed from the ACOX2 locus in breast cancers was identified (Additional file 1: Figure S1). This variant, ACOX2-i9, consists of exons 10–15 of the full-length transcript, with a start site in intron 9, approximately 150 base pairs upstream of exon 10 (Fig. 1b). Figure 1c shows the ACOX2-i9 sequence in a model together with the rat homolog, 2DDH, for which the crystal structure has been solved [15]. The ACOX2-i9 variant retains the sequence coding for a catalytic Acyl-CoA oxidase domain and the three amino acid C-terminal Peroxisomal Targeting Signal, but lacks the full flavin adenine dinucleotide (FAD) and fatty acid binding domain of the full-length protein [15]. ACOX2-i9 showed significantly higher expression in ER positive than in ER negative tumors (Additional file 1: Figure S1C, *p* = 0.0148). It is likely that the transcript detected in the breast tumors is the same variant that was described in hepatocellular carcinoma [12]. RNA-seq and clinical data from the publically available TCGA BRCA dataset was then used to validate the presence and expression of ACOX2-i9 [16]. The expression of ACOX2 and ACOX2-i9 in 87 tumor/normal pairs with known ER status was calculated from exon count data at the ACOX2 locus (see Methods for details). Plotting the difference in log2 ratio of ACOX2-i9 over ACOX2 in matched tumor/normal samples from TCGA confirmed that ACOX2-i9 has higher expression in tumors (Fig. 1d and Additional file 1: Figure S2) (Difference in log2 R(Tum)-log2 R(Norm) >1 in 82 of 87 samples). The full length ACOX2 transcript is expressed at higher levels in the normal samples from the same patient (Fig. 1e and Additional file 1: Figure S2B). ACOX2-i9 is expressed at significantly higher levels in ER-positive/Her2-negative samples compared to ER-negative/Her2-negative patients (Fig. 1f) (*p* = 0.000002, Wilcoxon rank sum test, ratio of ACOX2-i9/ACOX2), and is also higher in ER-negative/Her2-positive compared to ER-negative/Her2-negative patients (*p* = 0.001) (Fig. 1g).

### ACOX2-i9 is expressed in breast cancer cell lines

**Fig. 2:**
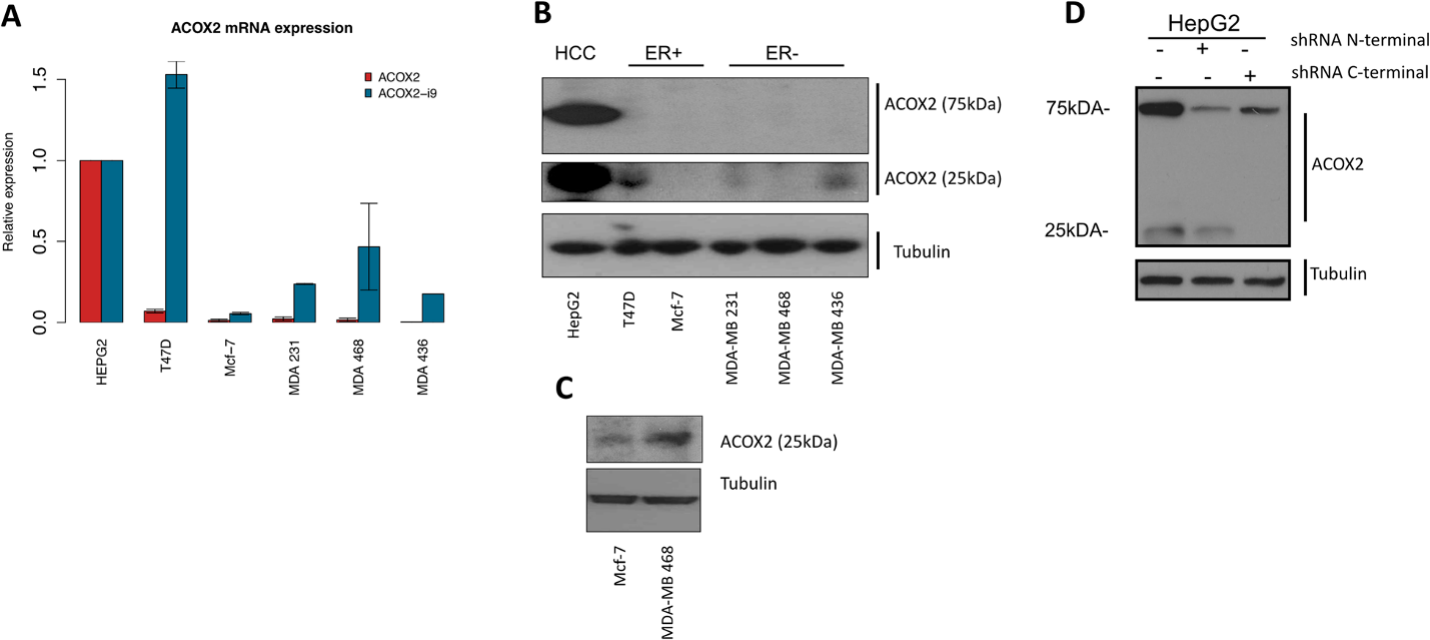
*Expression of ACOX2 in breast cancer cell lines*. ACOX2 and ACOX2-i9 mRNA levels were assessed by qRT-PCR in HepG2 cells and breast cancer cell lines (**a**), quantification is shown relative to HepG2 expression. Protein extracts from HepG2 and breast cancer cell lines were probed with a C-terminal antibody against ACOX2 (**b**). Highly sensitive chemiluminescent substrate (**c**) was included for illustration purposes to show even low levels of protein expression. HepG2 cells were transfected with shRNA targeting the N-terminal and C-terminal regions of ACOX2 (**d**)

RNA extracts from a panel of breast cancer cell lines, as well as HepG2 hepatocellular carcinoma cells, were analyzed for the full length and variant ACOX2 transcripts by qRT-PCR (Fig. 2a). Full length ACOX2 mRNA was mainly detected in the HepG2 and T47D cell lines, but it was also detectable in the MDA-MB 231 and MDA-MB 436 cell lines. Using a forward primer that hybridized to a region in intron 9 (see Methods for details) we observed that the variant transcript ACOX2-i9 was highly expressed in HepG2 and the ER positive T47D cell line, and present in detectable, but lower, levels in the ER positive Mcf-7 cell line, as well as in the ER negative MDA-MB 231, MDA-MB 436, and MDA-MB 468 cell lines.

### ACOX2-i9 is translated into protein

In order to identify a possible protein product of ACOX2-i9, protein was extracted from the breast cancer cell lines and HepG2 cells and probed with a monoclonal antibody raised against the far C-terminal (100aa) domain of ACOX2 (Fig. 2b). HepG2 cells showed high levels of full-lenght ACOX2 at 75 kDa, which was not detected in the breast cancer cell lines tested here. Instead, most prominently in T47D and MDA-MB 436, a faster migrating species was detected as 2 bands at approximately 25–30 kDa. This short variant was also strongly present in HepG2 cells, and detectable at low intensity in MDA-MB 321 cells. When a highly sensitive chemiluminescent substrate was used for photo-detection, Mcf-7 and MDA-MB 468 were also shown to express ACOX2-i9 (Fig. 2c).

In order to validate the protein product of ACOX2-i9, cDNAs encoding ACOX2 and ACOX2-i9 were engineered into expression vectors, both expressed in a cell free translation system and transfected into HeLa cells. Cells transfected with the full length ACOX2 showed a distinct band at 75 kDa, whereas expression of ACOX2-i9 gave rise to a double band at ~35 kDa, and when taking into account the presence of the 4 kDA tag, is consistent with the endogenous short form ACOX2 protein detected previously in the cell lines (Additional file 1: Figure S3A and B). The specificity of this 35 kDa protein was confirmed by successful knock down by introduction of an siRNA targeted to the C-terminal of ACOX2 (Additional file 1: Figure S3C).

shRNA constructs targeting the N- and C-terminal regions of ACOX2 were introduced to HepG2 cells to confirm that the lower molecular weight band was indeed an endogenous ACOX2 isoform (Fig. 2d). Cells expressing shRNA targeting the N-terminal end of ACOX2 showed reduced expression of the 75 kDa band. As expected, targeting the C-terminal end of ACOX2 both reduced the expression of the 75 kDa band, and eliminated the expression of the lower molecular weight bands. This shows that the low molecular weight band does indeed contain the C-terminal part of the ACOX2 transcript, and targeting this region significantly reduces the expression of the variant.

### Estrogen regulation of ACOX2-i9 in the T47D cell line

Data from chromatin immunoprecipitation sequencing (ChIP-seq) experiments of T47D cells showed an Estrogen Receptor binding peak in exon 10 of ACOX2 in the presence of Estradiol [17], and several consensus binding-sites for ESR1 were found in the sequence preceding exon 10 and within exon 10 itself (Additional file 1: Figure S4A).

**Fig. 3:**
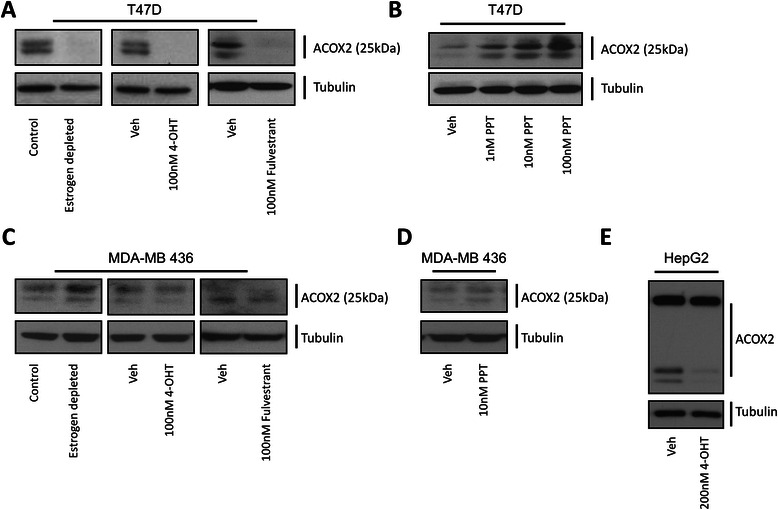
*Estrogen regulation of ACOX2-i9 in cell lines*. Western blot analysis was performed on whole cell lysates from T47D cells either depleted of estradiol for 72 h, treated with 4-OHT for 48 h, or treated with fulvestrant for 48 h (**a**), or treated with increasing doses of PPT for 48 h (**b**). MDA-MB 436 cells were either depleted of estradiol for 72 h, treated with 4-OHT for 48 h, or treated with fulvestrant for 48 h (**c**), or treated with PPT for 48 h (**d**), HepG2 cells were treated with 4-OHT for 48 h (**e**) (see Methods for details). Blots were probed with the C-terminal ACOX2 and Tubulin antibodies

To determine whether ACOX2-i9 protein expression is regulated by estrogens *in-vitro*, T47D cells were grown in estrogen depleted media (see Methods) for 72 h and protein extracts were probed with the ACOX2 C-terminal antibody. We found that expression of ACOX2-i9 was significantly down regulated (virtually abolished) in the absence of estrogen (Fig. 3a), The effect of treatment with several selective estrogen receptor modulators (SERMS) was then investigated. Treatment with the ER agonist/antagonist 4-OHT (tamoxifen) inhibited the expression of ACOX2-i9, an effect also observed when treating the cells with the selective estrogen receptor antagonist fulvestrant at 100nM, which is known to degrade ER protein expression. To determine if signaling by estrogen receptor alpha (ESR1) was the predominant mechanism, we stimulated T47D cells with the selective ESR1 agonist PPT. As shown in Fig. 3b, treatment with PPT increased ACOX2-i9 expression in T47D cells in a dose-dependent fashion. Similar effects on ACOX2-i9 expression were seen after estrogen depletion and tamoxifen treatments in the Mcf-7 cell line, but here the effect of fulvestrant was not prominent (Supplementary Figure S4B). PPT treatment also led to increased ACOX2-i9 expression.

To further demonstrate if the effect of these agents were indeed mediated through ESR1, the ESR1-negative MDA-MB-436 cells which express ACOX2-i9, were similarly treated. Here tamoxifen, fulvesterant, estrogen depletion, and PPT treatment led to no change in ACOX2-i9 expression, suggesting that in this cell line ACOX2-i9 is regulated in an estrogen-independent fashion (Fig. 3c and d).

Interestingly, in HepG2 cells, which express ESR1 [18] we could also observe decreased ACOX2-i9 expression upon treatment with tamoxifen (Fig. 3e), but these cells did not show reduced ACOX2-i9 expression in response to estrogen depletion (data not shown). Together these data indicate that the expression of ACOX2-i9 is effected by ESR1 stimulation and inhibition in breast cancer cell lines.

### Effect of ACOX2-i9 knock down on colony formation

**Fig. 4:**
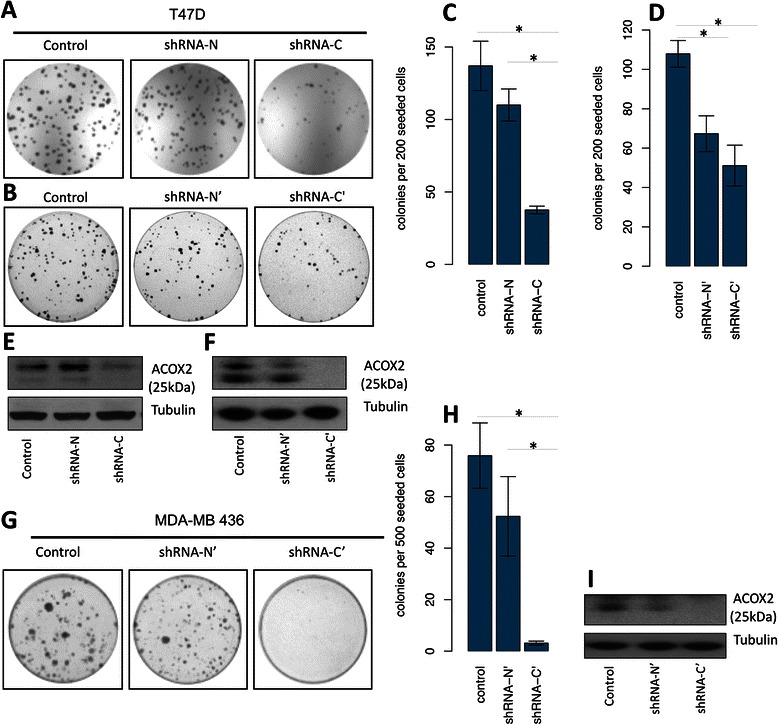
*ACOX2-i9 knockdown reduces growth of T47D and MDA-MB 436 cells*. Colony formation assay of T47D cells stably expressing shRNA targeting full length ACOX2 (shRNA N and N’), full length and ACOX2-i9 transcripts (shRNA C and C’), or empty vector (control) (**a** and **b**). Colony formation assay of MDA-MB 436 cells (**g**) using the N’, C’, and control constructs. Cells were methanol fixed and stained with Chrystal Violet. Colonies were counted manually (**c**, **d**, and **h**). Bars are average of three experiments performed in triplicates (+/− SE), *p < 0.05 as assed by two-sided *t*-test. Knockdown was assessed by Western blotting (**e**, **f**, and **i**)

ACOX2-i9 was found to be present in human breast cancer, but at low to zero levels in normal breast tissue samples. In order to investigate whether the presence of ACOX2-i9 gives a growth advantage to cells expressing the protein, short hairpin RNAs were stably introduced into T47D and MDA-MB 436 cell lines. Four different shRNA constructs were used, two complementary to the N-terminal region, (N and N’), targeting the full-length transcript of ACOX2, and two against the C-terminal, targeting both the full length and the short ACOX2-i9 transcripts (C and C’). A colony formation assay was carried out to assess whether knocking down ACOX2-i9 would affect the growth of T47D cells. Knockdown targeting the far C-terminal had a great effect on colony formation, reducing growth by 70 % and 50 % for C and C’ respectively (Fig. 4a, b, c, and d). The N-terminal shRNA construct (N) caused a slight but not significant reduction in colony formation while a second N-terminal construct reduced growth by approximately 35 % (N’). In MDA-MB 436 cells knockdown of both ACOX2 isoforms (C’) had a dramatic effect on colony formation, reducing growth by ~90 % (Fig. 4g and h). Knockdown of canonical ACOX2 (N’) did not cause a significant reduction in colonies. Knockdown was assessed by Western Blotting (Fig. 4e, f, and i). Although knockdown of canonical ACOX2 was not detectable at the protein level, both cell lines express mRNA transcripts detectable by qRT-PCR, and the slight effect on growth could be due to knockdown of ACOX2. The results suggest in ER+ and ER- cell lines that express ACOX2-i9, it is expressed as a functional protein involved in growth/proliferation of these cells in vitro.

### ACOX2-i9 expression is associated with better outcome in ER+ breast cancer

The clinical/biological relevance of this variant transcript of ACOX2 was further studied in an independent set of 113 breast tumor samples from patients from the well characterized MicMa cohort [14] for which long-term clinical follow up data were available. The variant transcript was first characterized by Sanger sequencing of a subset of the patient samples (*n* = 26), confirming that it includes a ~70 bp intronic sequence from intron 9 (data not shown). Following this, conventional RT-PCR was performed on RNA isolated from all 113 patient samples.

**Fig. 5:**
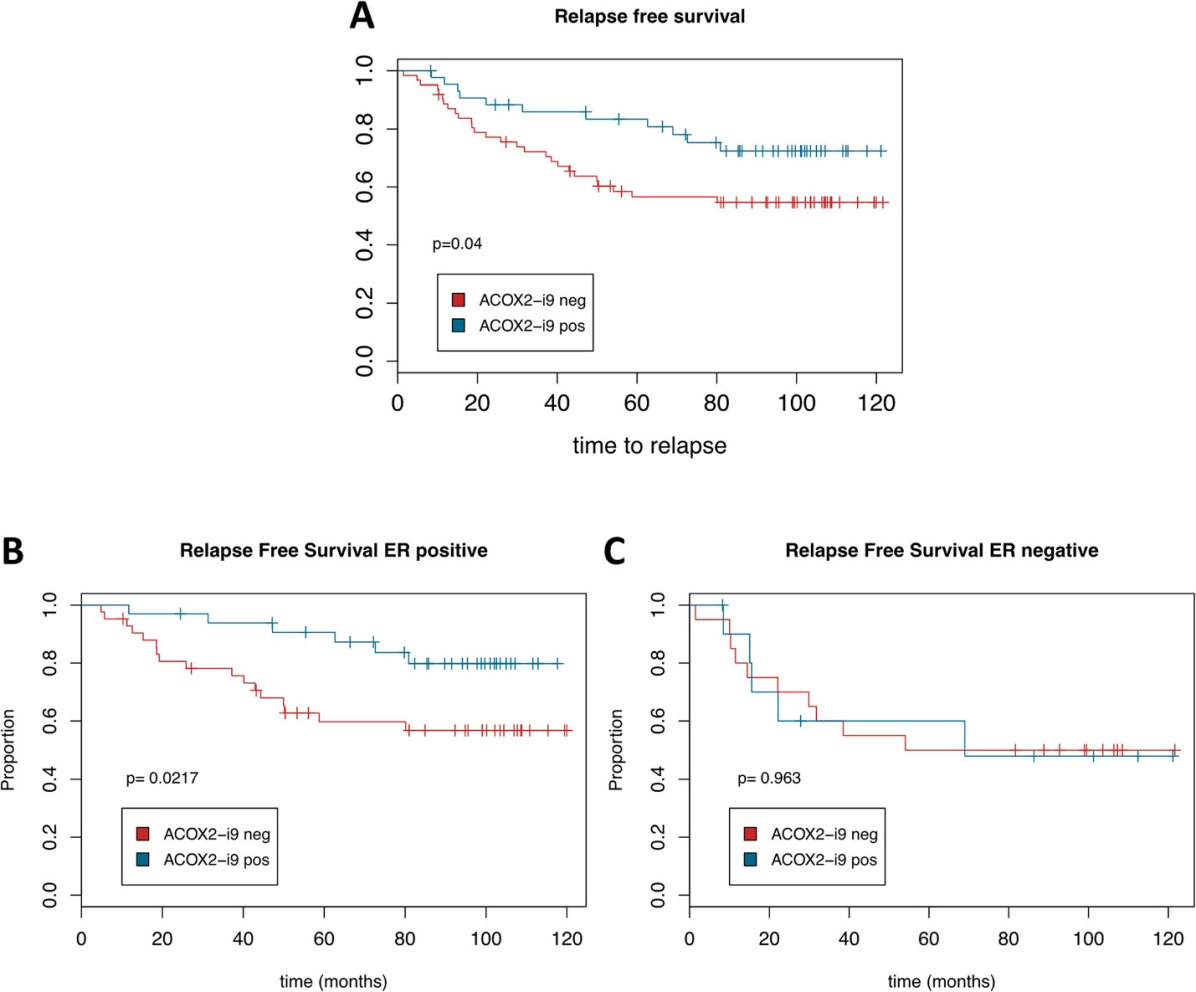
*ACOX2-i9 expression is associated with good prognosis in a cohort of breast cancer patients*. Kaplan-Meier survival curves of patients from the MicMa cohort testing positive (*n* = 44) or negative (*n* = 62) for ACOX2-i9 by PCR assay (**a**). **b** and **c** show survival curves for ER positive (ACOX2-i9pos *n* = 33, ACOX2-i9neg *n* = 42) and ER negative patients (ACOX2-i9pos *n* = 11, ACOX2-i9neg *n* = 20) respectively

Kaplan-Meier survival analysis for relapse free survival was performed on the patient cohort and ACOX2-i9 was a statistically significant predictor of outcome in this dataset, where presence of the variant transcript was associated with better outcome (*p* = 0.04) (Fig. 5a). Interestingly, this difference was strongly confined to the ER positive tumors, representing the subgroups that typically have a better prognosis than patients with ER negative tumors (*p* = 0.02) (Fig. 5b). Survival analysis of the ER negative patients showed that ACOX2-i9 had no effect on outcome (Fig. 5c). Further analysis showed that ACOX2-i9 expression associates with lower grade and p53 WT tumors, also within the ER+ patient group (Fisher exact test) (Tables 1 and 2). This is consistent with ACOX2-i9 expression in ER+ cancers being associated with luminal A breast cancer subtype.

**Table 1:** ACOX2-i9 association to clinical parameters in a cohort of 113 breast cancer patients

A										
		Tumor grade			ER		PR		TP53	
	N	1	2	3	Pos	Neg	Pos	Neg	WT	Mut
ACOX2-i9 pos	43	60 %	51 %	25 %	43 %	35 %	46 %	30 %	49 %	27.5 %
ACOX2-i9 neg	62	40 %	49 %	75 %	57 %	65 %	54 %	70 %	51 %	72.5 %
p value		0.015			0.52		0.14		0.04	

ACOX2-i9 was detected (pos) or not detected (neg) by PCR assay and correlated with tumor grade, estrogen receptor (ER), progesterone receptor (PR), or TP53 mutational status. A Fisher exact test was used to determine *P* values for the likelihood of association

**Table 2:** ACOX2-i9 association to clinical parameters within the ER+ patient group

B								
ER positive		Tumor grade			PR		TP53	
	N	1	2	3	Pos	Neg	WT	Mut
ACOX2-i9 pos	33	60 %	51 %	20 %	47 %	11 %	51 %	21 %
ACOX2-i9 neg	43	40 %	49 %	80 %	53 %	89 %	49 %	79 %
p value		0.015			0.07		0.03	

## Discussion

In this study we have shown that a variant (shorter) transcript of ACOX2, identified by RNA sequencing, translates into a protein detectable in several breast cancer cell lines, as well as the HCC cell line, HepG2. When the ACOX2-i9 transcript was expressed in HeLa cells the protein lysate probed with the C-terminal ACOX2 antibody gave rise to two bands at ~35 kDa. *In Vitro* translation of the same construct in a cell free system also resulted in two bands of this size, indicating that the transcript might harbor more than one transcription start site. The size of the bands at ~35 kDa including a 4 kDa molecular tag is slightly higher than that of the endogenous ~25 kDa bands observed in the cell lines. The translational start site could be located downstream of the 5’ transcript sequence observed by RNA sequencing, or the endogenous protein could be subject to post-translational modifications. Nonetheless, the 25 kDa protein present in the cell lines was detected by an ACOX2 antibody that recognizes the far C-terminal part of the protein, and is very likely to include the Acyl-CoA oxidase domain and the Peroxisomal Targeting Signal. Previously we, and others [19] have shown that this variant is virtually absent in normal breast tissue samples. In 2010 Hodo et al. showed the presence of an intronic start variant of ACOX2 in HCC [12] which is likely to be the same as ACOX2-i9. They reported that the expression of the intronic variant is associated with moderately differentiated tumors, and could be involved in HCC tumor progression.

We identified ACOX2-i9 as a transcript expressed at higher levels in ER+ than in ER- breast carcinoma patients. The sequence preceding the intronic start site contains several ESR1 canonical binding sites, and an ESR1 peak was observed in exon 10 of ACOX2 in a ChIP-seq analysis in T47D cells as reported by Joseph et al. [17]. We observed reduced expression of ACOX2-i9 both upon depleting the cells of estrogen, and by treating the cells with the known ER agonist/antagonist tamoxifen and the selective estrogen receptor antagonist fulvestrant, as well as induction of ACOX2-i9 protein when treated with ESR1 agonist PPT. These interventions did not affect ACOX2-i9 levels in an ER- breast cancer cell line that did express ACOX2-i9, confirming that the effects of these agents in ER+ cells is likely mediated through ESR1.

Although ACOX2-i9 showed overall higher expression in ER+ patients it is clearly expressed in a subset of ER- patients, and in ER- cell lines as shown above. Regulation of ACOX2-i9 appears to be estrogen-independent in ER- cell lines. Transcription factors such as Jun, Fos, and SP-1 have been shown to bind in the region preceding exon 10 of ACOX2 (ENCODE), and are possible regulators of expression in the ER- cell line, but the exact mechanism is not clear at this point. Interestingly the HCC cell line HepG2, which also express ESR1, also responded to tamoxifen by down-regulating ACOX2-i9 expression. Even though ACOX2-i9 expression in these cells is regulated by tamoxifen, canonical ACOX2 is not, a clear example of separate transcripts from the same gene locus being under individual control.

ACOX2-i9 consists of ~300 amino acids in the C-terminal region of the canonical ACOX2. This isoform contains the Acyl-CoA oxidase domain, but lacks the FAD binding domain. To determine whether the ACOX2-i9 isoform is functional we knocked down its expression using shRNA constructs in T47D and MDA-MB 436 cells. Knocking down the canonical ACOX2 had a modest effect on both T47D and MDA-MB 436 cells. Knockdown targeting the C-terminal region dramatically reduced colony formation in both cell lines, indicating that ACOX2-i9, when expressed, is involved in growth/proliferation in both ER+ and ER- cells *in-vitro*.

We verified the expression of ACOX2-i9 in the TCGA breast cancer cohort and in an independent group of breast cancer patients with long term follow up. Interestingly, in the patients positive for ACOX2-i9, the expression of the variant was highly confined to the tumor, and low or not present in the adjacent normal sample. The full length ACOX2 transcript was expressed at higher levels in the majority of the normal samples. Expression levels of each exon in the ACOX2 locus showed that the last 5 exons (ACOX2-i9) were higher in ER+/Her2- compared to ER-/Her2- patients in the TCGA cohort, and in the ER-/Her2+ patient group compared to ER-/Her2- patients. In the patients with long term follow up ACOX2-i9 expression was assayed by conventional PCR, and the ratio to the full-length transcript could not be determined.

Detection of the variant by this method was associated with good prognosis in the patient cohort as a whole. The prognostic association with ACOX2-i9 expression was limited to ER+ cancers, with no such association seen in ER- cancers. As most ER+ cancers in this cohort were treated with hormonal therapy, it is possible that ACOX2-i9 expression may be associated with sensitivity to this treatment. Alternatively, ACOX2-i9 expression may mark a set of better prognosis ER+ cancers (ie luminal A breast cancers) regardless of hormonal therapy.

ACOX2-i9 knockdown resulted in less growth/proliferation of cell lines as shown here by colony formation assays, suggesting the ACOX2-i9 expression is required for optimal growth. These findings raise the possibility that ACOX2-i9 may both be a prognostic marker and a potential therapeutic target. Although it may seem contradictory at first that a gene positively involved in tumor growth can be associated with improved outcome, there are several examples of this in the literature and clinic. In a heterogenous set of cancers, such as breast cancer, with many subclasses, a gene may both be a potent growth driver and mark a class of tumors that do relatively better. A relevant example is high ESR1 expression, which is associated with good prognosis in breast cancer (and marks the Luminal A subclass), but is required for growth and is an excellent therapeutic target [20–24]. Within ER+ breast cancer higher expression of ESR1 is also associated with good prognosis [25, 26]. As ACOX2-i9 is estrogen regulated in ER+ cancer cells, it is not surprising that high levels may be associated with estrogen-responsiveness and good prognosis in the ER+ patient group. However as even “good prognosis” subset of ER+ cancers have a significant recurrence rate with standard hormonal therapy, there is a clear need to find other novel potential therapeutic targets in these cancers.

The secondary bile acid Chenodeoxycholic acid (CDCA), the end product of the bile acid pathway in which ACOX2 functions, has been shown to stimulate proliferation in ER+ cells [27], and may contribute to the effects seen of ACOX2 on proliferation. The cholesterol metabolite, 27-hydroxycholesterol, was recently shown to increase ER-dependent growth in breast cancer cell lines and mouse models [28]. High expression of the enzyme that catalyzes the conversion of cholesterol to 27HC, CYP27A1, is associated with higher tumor grade in breast cancers, and high expression of CYP7B1, the enzyme downstream of CYP27A1 is associated with better outcome in ER+ breast cancer patients. ACOX2-i9, which we show is associated with better outcome in ER+ patients, could be involved in the downstream catabolism of 27HC. The interplay between these two pathways can possibly shed light on the opposing roles of ACOX2 in tumorigenesis.

Of note, tamoxifen treatment specifically reduced expression of the ACOX2-i9 variant in HepG2 hepatic cells, while having no effect on the full length ACOX2. As tamoxifen is known to induce fatty liver [29], this observation raises the possibility that tamoxifen induced regulation of the ACOX2 variant may affect fatty acid metabolism in the liver. Estrogen-regulation of fatty acid metabolism has been observed in several clinical settings [30–32], and it is possible that estrogen regulation of ACOX2 variant transcripts could contribute to this process.

Future investigation is necessary to determine whether the substrate specificity of ACOX2-i9 is different from that of canonical ACOX2. As this variant transcript is represented in all the clinical breast cancer subgroups, and mainly confined to the tumor, ACOX2-i9 could be a possible independent target for therapy.

## Conclusions

In this study we show that the alternate start transcript translates into a 25 kDa protein and is present in several breast cancer cell lines, as well as the HCC cell line HepG2. The expression of this ACOX2 isoform is positively regulated by PPT, and down regulated by the ER agonist/antagonist tamoxifen and fulvestrant. Knockdown of ACOX2-i9 leads to impaired cell growth of both T47D and MDA-MB 436 cells. These data suggest that ACOX2-i9 is required for optimal growth of ER+ breast cancer and may be a novel therapeutic target.

## Additional file

Additional file 1: **Figures S1-S3 are supplied in Additional_File_1.pdf and includes figures showing Visualization of RNA sequencing reads in the ACOX2 locus (S1), additional figures supplementing Figure** 1**, showing expression of ACOX2-i9 in tumor/normal pairs in the TCGA data (S2).** S3 supplements Figure 3, showing additional cloning, expression and knockdown of the ACOX2-i9 protein in cell lines. Additional agonist/antagonist treatment results from the Mcf-7 cell line are shown in S4.

## Abbreviations

ACOX2: Acyl-CoA oxidase 2
RNA-seq: Ribonucleic acid-sequencing
ER: Estrogen Receptor
TCGA: The Cancer Genome Atlas
BRCA: Breast cancer dataset (from TCGA)
qRT-PCR: Quantitative reverse transcriptase-polymerase chain reaction
shRNA: short hairpin-RNA
FAO: Fatty acid oxidation
HCC: Hepatocellular carcinoma
E2: 17-β-Estradiol
4-OHT: 4-Hydroxytamoxifen
RPKM: Reads per kilobase of transcript per million reads mapped
FAD: Flavin adenine dinucleotide
ChIP-seq: Chromatin immunoprecipitation sequencing
VLCFA: Very long chain fatty acids
BCFA: Branched chain fatty acids
PPT: Propyl pyrazoletriol
CDCA: Chenodeoxycholic acid
SERM: Selective estrogen receptor modulator
