# Supplementary material for: Expression of an estrogen-regulated variant transcript of the peroxisomal branched chain fatty acid oxidase ACOX2 in breast carcinomas
Source: BMC Cancer. 2015 Jul 17;15:524. doi: 10.1186/s12885-015-1510-8 (PMC4504068; doi:10.1186/s12885-015-1510-8)
Supplement: Additional file 1: — Figures S1-S3 are supplied in Additional_File_1.pdf and includes figures showing Visualization of RNA sequencing reads in the ACOX2 locus (S1), additional figures supplementing Figure 1, showing expression of ACOX2-i9 in tumor/normal pairs in the TCGA data (S2). S3 supplements Figure 3, showing additional cloning, expression and knockdown of the ACOX2-i9 protein in cell lines. Additional agonist/antagonist treatment results from the Mcf-7 cell line are shown in S4. [file 12885_2015_1510_MOESM1_ESM.pdf]

Supplementary Figure 1

A

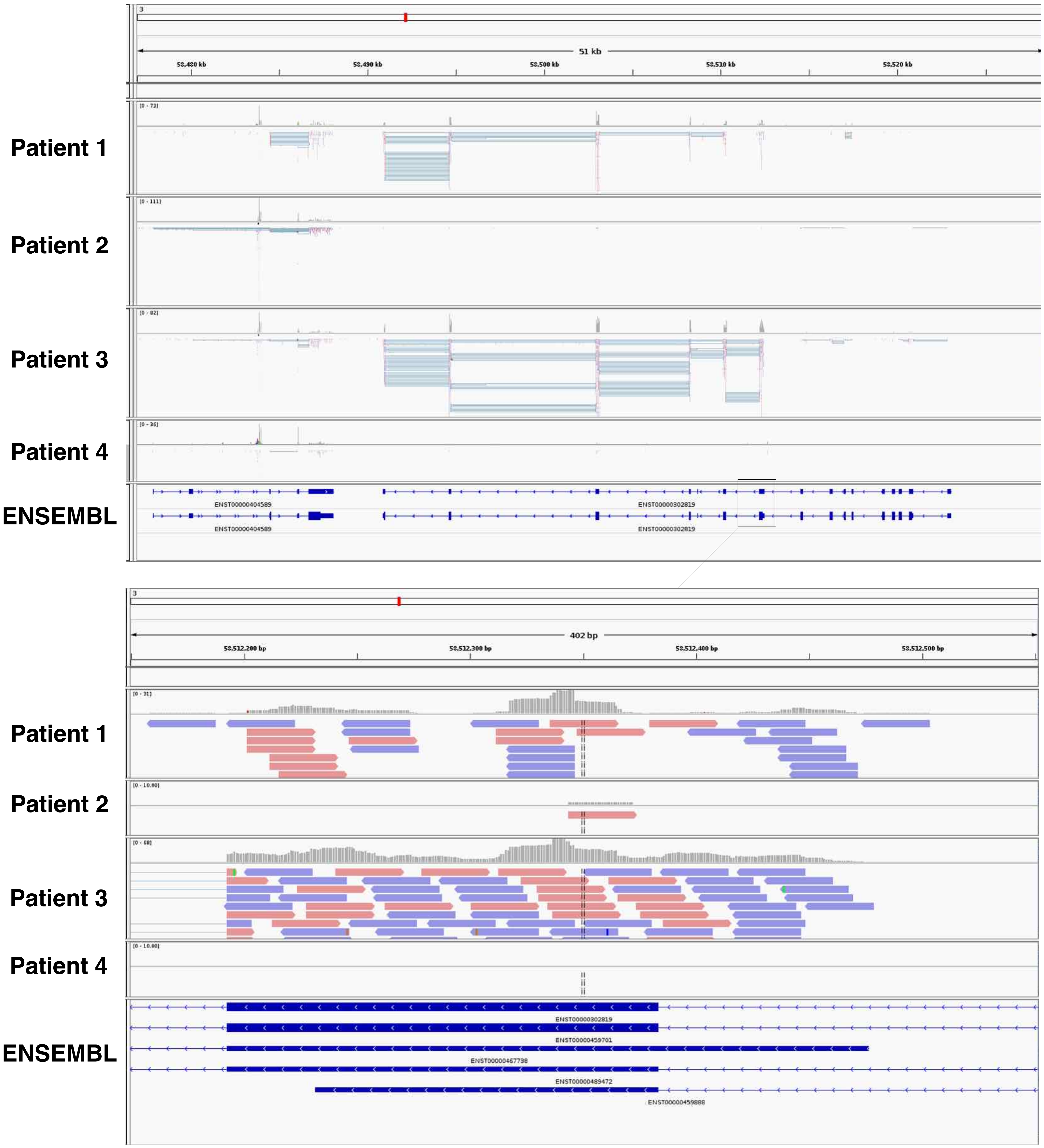

B

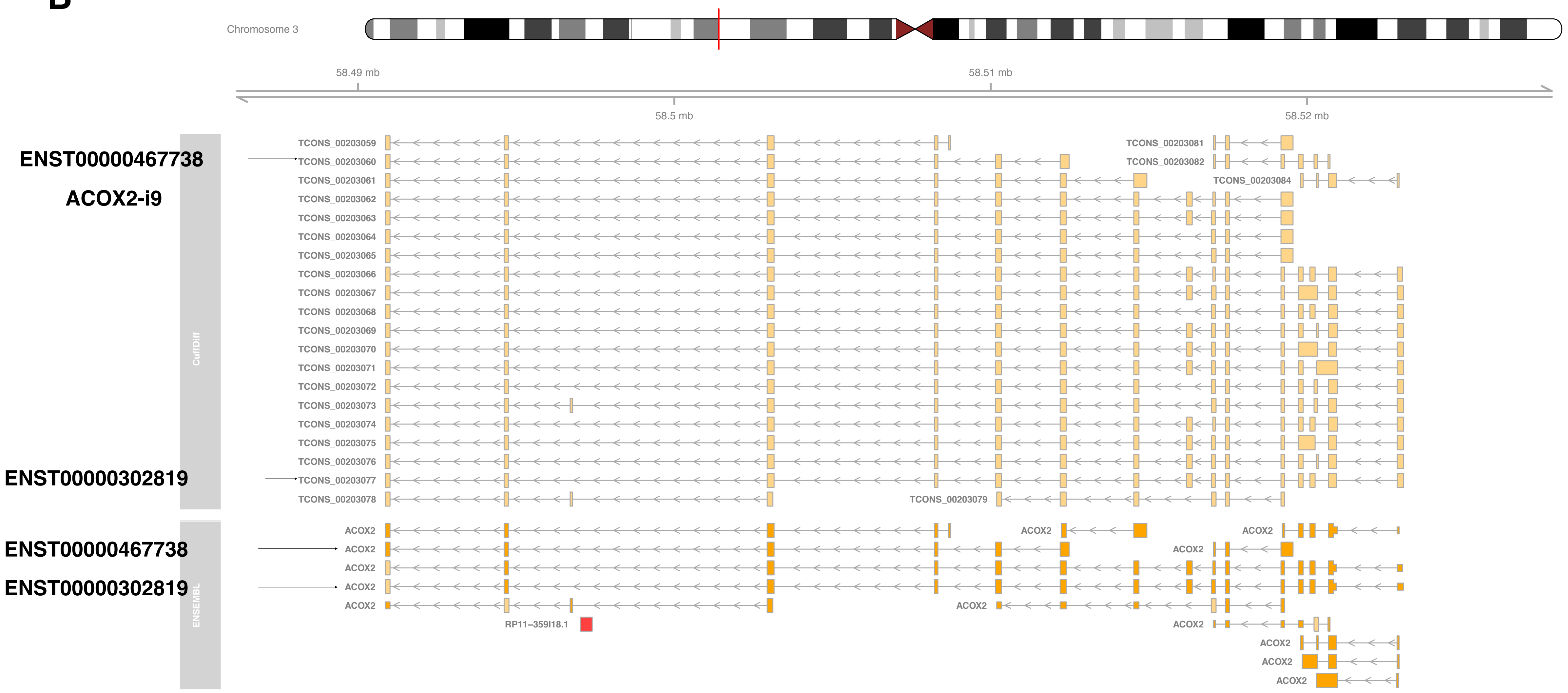

C

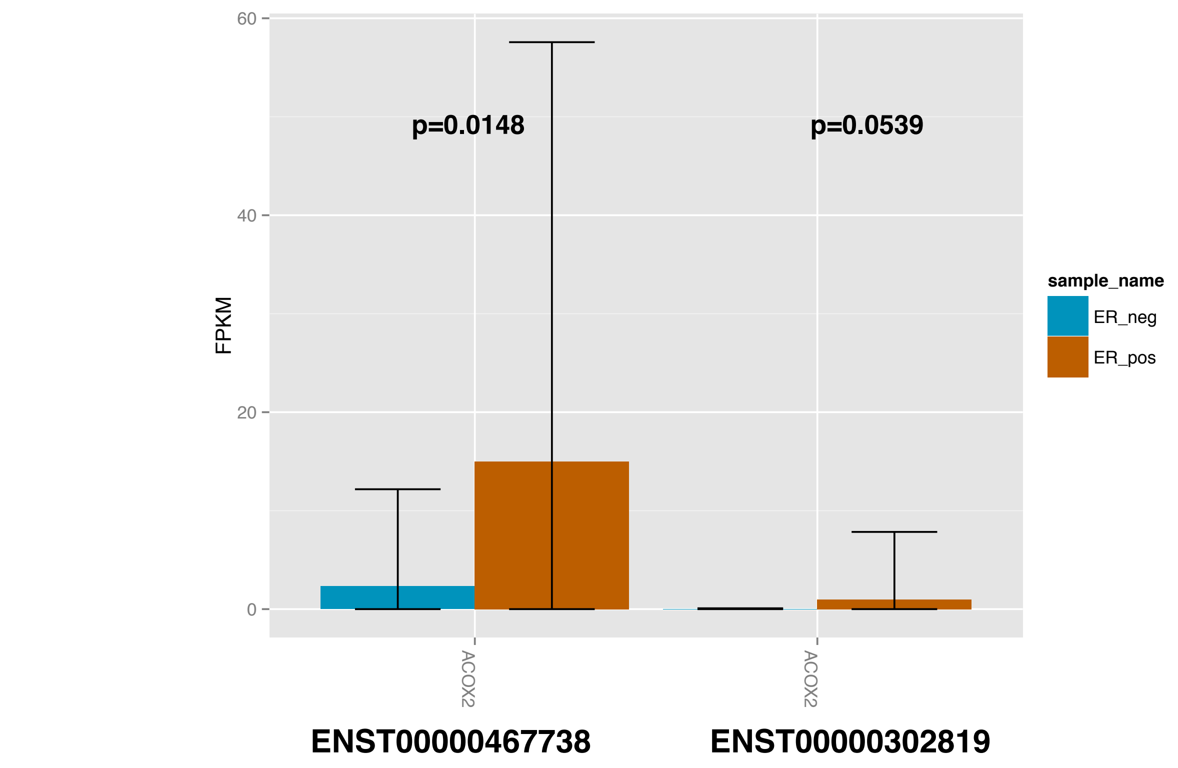

Supplementary Figure S1. *Visualization of RNA sequencing reads in the ACOX2 locus*. RNA sequencing reads mapped to the human genome from four selected patients was visualized with IGV, and annotated with a genome track from ENSEMBL (hg19)(A). Zoomed in to exon 10 of ACOX2 reads were shown to be present upstream in intron 9, in two of the patients shown here. A gene model of ACOX2 built with Cufflinks identified the transcript ENST00000467738, ACOX2-i9, as present in the dataset (B). FPKM expression of ENST00000467738 (ACOX2-i9) and the full length ACOX2, ENST00000302819 in ER positive and ER negative patients is shown in C. More analysis of the RNA-seq dataset is presented in Seiler et al. (Submitted manuscript).

Supplementary Figure 2

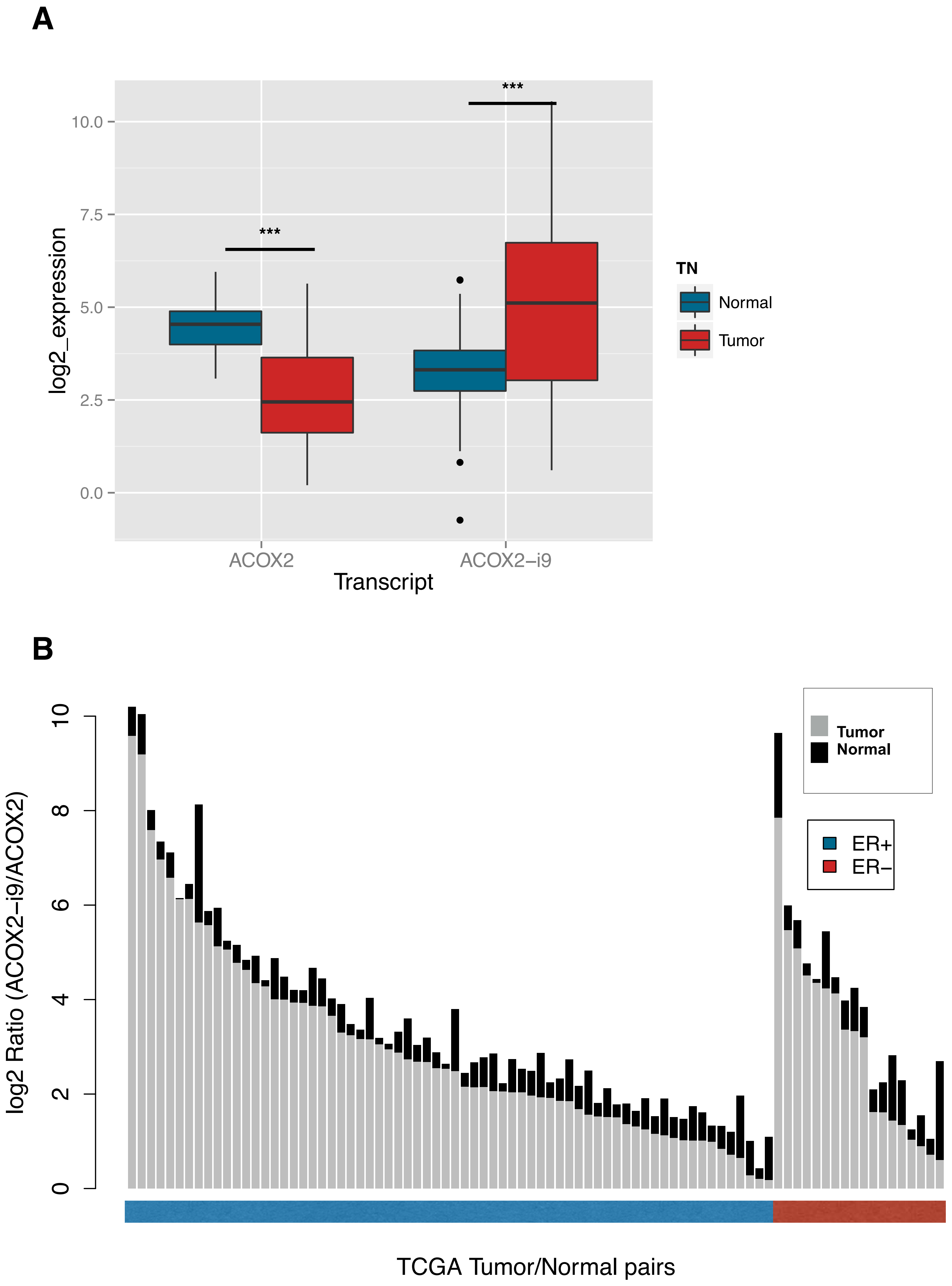

Supplementary Figure S2. *Expression of ACOX2 and ACOX2-i9 in TCGA BRCA tumor/normal pairs*. Log2 expression levels of ACOX2 (F0, see methods for details) and ACOX2-i9 (F1) in tumor/normal pairs from 87 patients in the TCGA BRCA dataset as measured by RNA-seq. A significant difference is seen both in the ACOX2 expression, which is higher in normal samples than in tumor (P=2.2e-16, Wilcoxon rank sum test), and in ACOX2-i9 which is expressed at higher levels in tumor compared to normal samples (p=4.299e-08)(A). The log2 Ratio of ACOX2-i9/ACOX2 for each individual patient pair is shown in B.

Supplementary Figure 3

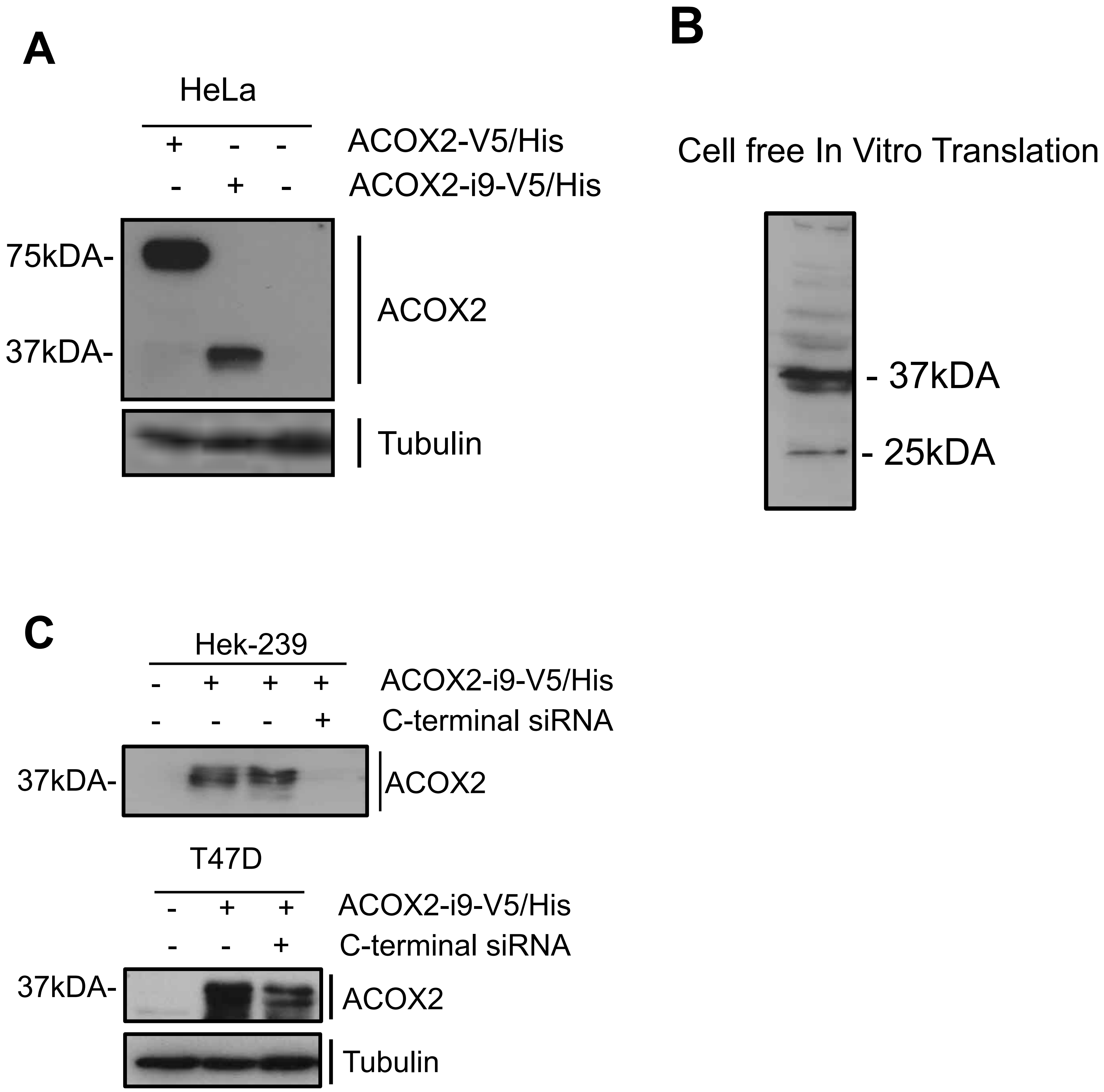

Supplementary Figure S3. *Expression of ACOX2 and ACOX2-i9 in-vitro*. HeLa cells were transfected with pcDNA3.1-V5/His empty vector, or vector containing the ACOX2 or ACOX2-i9 transcripts (A). ACOX2-i9 tagged with V5/His was expressed in a cell free In vitro environment (B). Hek-293 and T47D cells were transfected with pcDNA3.1-ACOX2-i9-V5/His and siRNA targeting the C-terminal region of ACOX2 (C). All Protein lysates were probed with the C-terminal ACOX2 and Tubulin antibodies.

Supplementary Figure 4

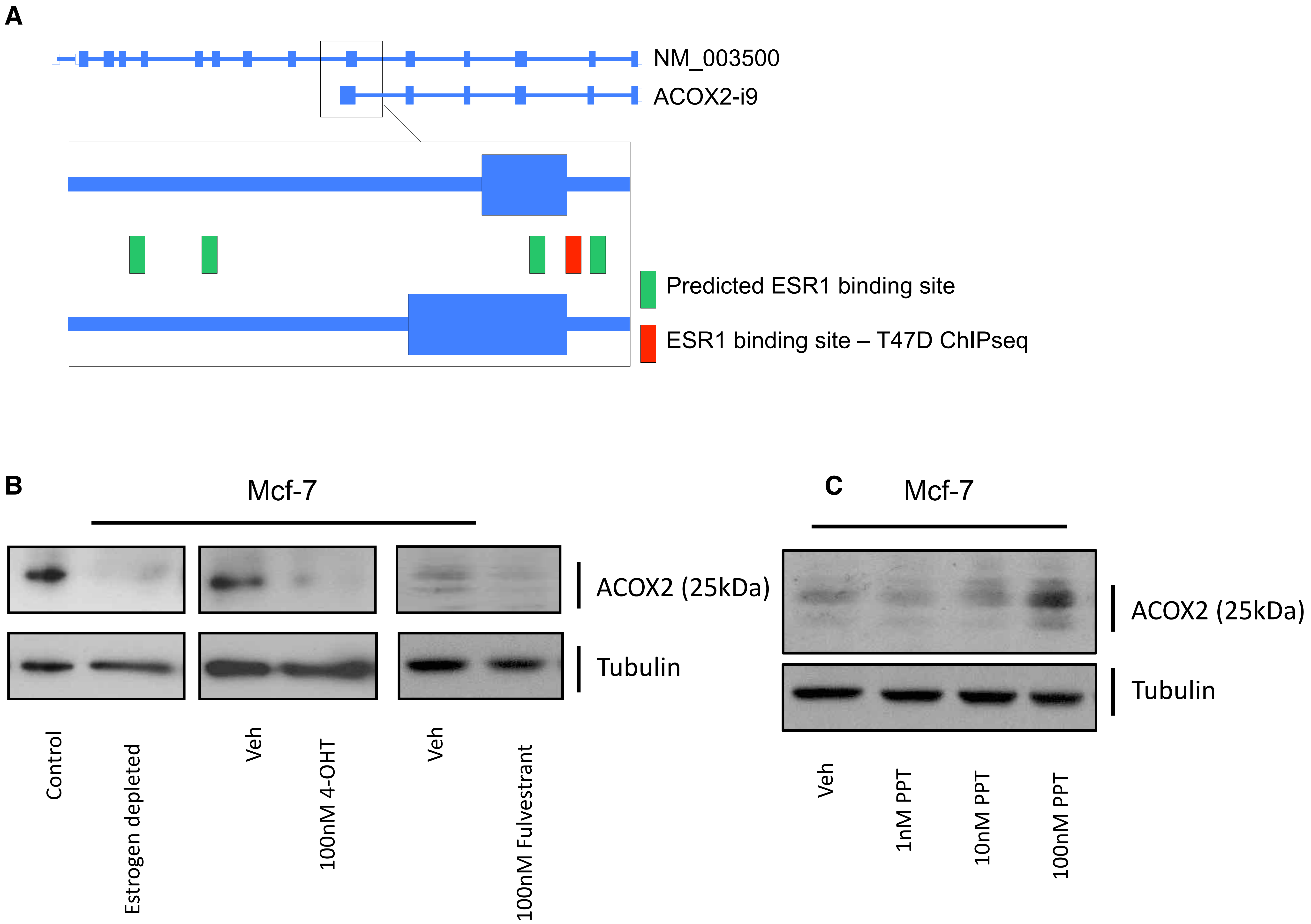

Supplementary Figure S4. *Estrogen regulation of ACOX2-i9 in cell lines.* Figure S4A shows an ER binding site in T47D cells as reported by [17] in addition to predicted ER binding sites in vicinity to the ACOX2-i9 transcription start site. Western blot analysis was performed on whole cell lysates from Mcf-7 cells either depleted of estradiol for 72h, treated with 4-OHT for 48h, or treated with fulvestrant for 48h (B), or treated with increasing doses of PPT for 48h (C).
